# Microbial Contamination of Radiology Equipment: A Descriptive Study in Ghana

**DOI:** 10.1002/hsr2.73326

**Published:** 2026-09-30

**Authors:** Seth Kwadjo Angmorterh, John Gameli Deku, Emmanuel Akyea‐Mensah, Riaan Van de Venter, Hubert Amu, Sonia Aboagye, Patience Nyamekye Agyemang, Nathaniel Awentiirin Angaag, Jevas Omweri Kenyanya, Kafui Kossi Kekessie, Benjamin Klugah‐Brown

**Affiliations:** ^1^ Department of Medical Imaging, School of Allied Health Sciences University of Health and Allied Sciences Ho Ghana; ^2^ Department of Medical Laboratory Sciences, School of Allied Health Sciences University of Health and Allied Sciences Ho Ghana; ^3^ Department of Radiography, School of Clinical Care and Medicinal Sciences, Faculty of Health Sciences Nelson Mandela University Gqeberha South Africa; ^4^ Department of Population and Behavioural Sciences, Fred N. Binka School of Public Health University of Health and Allied Sciences Hohoe Ghana; ^5^ Department of Speech, Language & Hearing Sciences, School of Allied Health Sciences University of Health and Allied Sciences Ho Ghana; ^6^ The Clinical Hospital of Chengdu Brain Science Institute, MOE Key Laboratory for Neuroinformation, School of Life Science and Technology University of Electronic Science and Technology of China Chengdu Sichuan China

**Keywords:** Ghana, hospital‐acquired infections, infection prevention and control, nosocomial pathogens, patient safety, radiography, radiology

## Abstract

**Background and Aims:**

Nosocomial infections continue to impose significant clinical and economic burdens on patients and healthcare systems worldwide. Radiology equipment, accessories, and work surfaces can become contaminated with healthcare‐associated pathogens due to frequent handling by healthcare personnel and patients. This study investigated microbial contamination on commonly used radiology equipment and surfaces at a major hospital in Ghana.

**Methods:**

This descriptive microbiological study involved weekly swabbing of 10 radiology equipment/accessories and surfaces using sterile nylon swabs over a 4‐week period. Swab samples were transferred into bijoux bottles containing peptone broth to enhance microbial growth. For bacterial isolation, samples were inoculated onto MacConkey and Blood Agar plates and incubated appropriately. Bacterial isolates were identified based on colonial morphology, Gram staining, and biochemical reactions. Fungal cultures were performed using Sabouraud Dextrose Agar and incubated at 28°C for 7 days. Fungal isolates were identified using colonial morphology, tease mount preparation, and lactophenol cotton blue staining.

**Results:**

Microbial contamination was detected in 35 (87.5%) of the 40 samples analyzed. Identified bacterial isolates included coagulase‐negative Staphylococci, *Klebsiella pneumoniae, Bacillus* species, and *Staphylococcus aureus*. These organisms were isolated from the 35/43 cm cassette, x‐ray tube head handle, horizontal bucky surface, and erect bucky surface. The highest bacterial colony count recorded was 1.2 × 10^6 ^CFU/cm^2^. The 35/43 cm x‐ray cassette demonstrated the highest level of contamination, accounting for 4 of the 9 bacterial isolates identified. Fungal isolates, including *Neurospora* species and *Fusarium solani*, were also detected.

**Conclusion:**

Radiology equipment and surfaces harbored potentially pathogenic bacterial and fungal organisms, indicating their capacity to act as environmental reservoirs within radiology departments. The detection of both bacterial and fungal species on frequently touched radiology items highlights the need for strict adherence to infection prevention and control protocols to enhance the safety of both patients and healthcare professionals.

## 1 Introduction

Nosocomial infections are infections acquired during hospital visits that are secondary to a patient's original condition [1]. Globally, these infections impose substantial clinical and economic burdens on healthcare systems. For instance, the United States of America (USA) spends between $96 and $147 billion annually on their management [2], while the annual financial cost in sub‐Saharan Africa is estimated at $13 billion [3]. Nosocomial infections increase both morbidity and mortality, particularly among vulnerable populations, including pediatric, elderly, pregnant, and immunocompromised patients, as well as individuals with chronic diseases [4, 5, 6].

Radiology equipment, accessories, and work surfaces, such as image receptors, x‐ray tables, and door handles, are potential fomites that can harbor and transmit nosocomial pathogens [7, 8, 9]. This risk is amplified by the high daily patient throughput in radiology departments, where individuals with diverse health conditions share equipment and clinical spaces. Inadequate staffing, limited resources, and insufficient knowledge of infection prevention and control (IPC) practices may further compromise decontamination procedures and increase the risk of infection transmission. In low‐ and middle‐income countries (LMICs), including Ghana, this problem may be more pronounced because of mismatches between available equipment, staffing levels, and patient throughput [10, 11, 12]. During most projectional radiography procedures, direct contact occurs among radiographers, patients, and imaging equipment, potentially facilitating microbial transmission. Previous studies have demonstrated contamination of radiology accessories, including x‐ray tube handles, control panels, ultrasound probes, and image receptors, with various microorganisms that may serve as potential sources of healthcare‐associated infections [13, 14].

Several international studies have also reported contamination within radiology departments. Investigations in Ireland [15] and South Korea [7] reported that the bore of magnetic resonance imaging (MRI) scanners and image receptors used in projectional radiography were contaminated with methicillin‐resistant *Staphylococcus aureus*. Harvin et al. [8] in the USA also indicated that the exposure and contrast administration buttons of various radiology equipment had high levels of *S. aureus* contamination. Similarly, the vast majority of image receptors in a radiology department in the United Kingdom (UK) showed high levels of *S. aureus*, *Micrococcus luteus*, coagulase‐negative Staphylococcus, and diphtheroids [16]. In Nigeria, Oshoma et al. [9] found that image receptors and the compression paddle of the mammography equipment were highly contaminated with microbes and could serve as vectors for nosocomial infections. In Ghana, Adomako et al. [17] investigated the presence of pathogens on radiology equipment and accessories at a major hospital in Accra (the capital of Ghana) and found that microbes were present on radiology equipment and accessories and served as vehicles for nosocomial pathogens.

To the best of our knowledge, no previous study has systematically investigated microbial contamination of radiology equipment, accessories, and surfaces in a large hospital within Ghana's Volta Region. The present study, therefore, aimed to determine the presence and distribution of microorganisms on radiology equipment, accessories, and work surfaces at a major hospital in the Volta Region of Ghana. These findings provide locally relevant evidence to inform and strengthen IPC protocols within radiology departments and improve patient and staff safety.

## 2 Method

Ethical approval was obtained from the Research Ethics Committee of the University of Health and Allied Sciences (UHAS‐REC A.8 [124] 22–23). Informed consent was not required as the study did not involve human participants or identifiable data.

### 2.1 Study Setting

This study was conducted in the Radiology Department of a major hospital in the Volta Region of Ghana. The 300‐bed hospital provides a wide range of clinical services, including outpatient, inpatient, mental health, dialysis, orthopedic, and radiology care. It was selected because it houses the largest radiology unit in the region and serves patients from surrounding districts and neighboring countries, primarily Togo and Benin. The department is equipped with projectional radiography, ultrasound, fluoroscopy, and computed tomography (CT) systems. It operates a 12‐h daily service, staffed by eight radiographers, two radiologists, and three support personnel, and attends to approximately 70 in‐patients and out‐patients daily.

### 2.2 Study Design

The study employed a descriptive microbiological research design. This study design was adopted to characterize the presence, type, and distribution of microbial contamination on radiology equipment, accessories, and surfaces under normal, unaltered conditions.

### 2.3 Radiology Equipment, Accessories and Working Surfaces

The details of the radiology equipment, accessories, and working surfaces that were swabbed are shown in Table 1. These surfaces were selected because they are most frequently used in the department, and previous studies have also shown that they are the most prone to contamination. These surfaces are likely hotspots for the spread of germs, so focusing on them will help reduce transmission risk. For example, the main door handle is frequently touched by many individuals and can easily transfer germs among people. Also, the erect and horizontal Bucky handles and surfaces, tube head collimator knob, x‐ray tube head handle, exposure button and the image receptors are also high‐touch surfaces that can harbor microbes. By focusing on these surfaces, studies can better understand the risks of contamination and develop strategies to reduce the spread of germs.

**Table 1:** Radiology equipment, accessories, and surfaces screened for microbes.

Number	Equipment/accessories/surface	Number of swabs
1	Main door handle	One swab per week per radiology equipment, accessory, and work surface for 4 weeks
2	Erect Bucky surface	
3	Erect Bucky handle	
4	Horizontal Bucky surface	
5	Horizontal Bucky handle	
6	Tube head collimator knob	
7	X‐ray tube head handle	
8	Exposure button	
9	24/30 cm Image receptor	
10	35/43 cm Image receptor	
Total		40

### 2.4 Radiology Surfaces Swabbing Process

Each of the surfaces was randomly swabbed four times. The swabs were done once per week for a 4‐week period. This resulted in a total of 40 swab samples. No prior warnings of the swabs were given to the radiology staff, and routine decontamination protocols of the radiology department were not altered in any way prior to this study. These measures helped to minimize the likelihood of the Hawthorne effect on the radiology staff, where there is behavior change as a result of knowing one is being observed [18]. Routine cleaning of the equipment, accessories, and work surfaces was performed before the commencement of daily clinical activities by the designated cleaning staff. A 0.5% sodium hypochlorite solution, prepared by diluting a 5% sodium hypochlorite (bleach) stock solution at a ratio of 1:9 with water, with a small amount of Bine 20 added, was used for routine disinfection. Thereafter, intermittent cleaning was carried out only when surfaces became visibly soiled following patient examinations. Sterile swab samples were collected during routine working hours, under normal clinical operating conditions, rather than immediately after scheduled cleaning. The swabbing was performed by a clinical microbiologist with over 15 years of work experience. Sterile hand gloves were donned before swabbing to prevent cross‐contamination, and the gloves were replaced after each swabbing. The entire 2 cm^2^ area was swabbed by applying consistent moderate pressure, equivalent to the pressure used to write with a ballpoint pen, using approximately 10 horizontal and 10 vertical passes. The swabs were rotated during the process to maximize contact with the surfaces. The swabbing was performed by the same microbiologist to ensure consistency across samples and reduce operator‐dependent variations. The swabbed samples from each surface were placed in bijou bottles with peptone broth to promote growth. Each bijou bottle was labeled with the name of the equipment, accessory, or surface for easy identification. The sterile swabs were transported to a microbiology laboratory for culturing.

### 2.5 Laboratory Procedures and Analysis

The swabs in the peptone broth were vortexed, and serial dilutions were made to determine colony count by the pour plate method using Plate Count Agar, according to Sanders [19]. Incubation was done at 37°C for 18–24 h. Colonies between 30 and 300 were counted and expressed as CFU/cm^2^ using the existing formula by Lugbe et al. [20]:

$${\text{CFU}/\text{cm}}^{2}=\frac{\text{Number}\;\text{of}\;\text{colonies counted}\;\times \;\text{Dilution}\;\text{factor}\times \text{Total}\;\text{elution}\;\text{volume }\left(\text{mL}\right)}{\text{Volume}\;\text{plated }\left(\text{mL}\right)\times \text{Area}\;\text{swabbed }\left({\text{cm}}^{2}\right)}$$

where the total elusion volume was 10 mL and area swabbed was 2 cm^2^.

Growth below the countable range (< 30 colonies) was considered statistically non‐significant colonies. The swabs were cultured on both MacConkey and blood agar plates, followed by an incubation period of 18–24 h aerobically at 37°C, as done in previous studies [13, 17]. Identification was done by describing the colonial morphology on the agar plates, Gram staining, motility testing, and by using biochemical tests, including catalase, coagulase, oxidase, urease, citrate, indole, and triple sugar fermentation tests according to the method employed by Deku et al. [21]. *E. coli* ATCC 25922 and *K. pneumoniae* ATCC 700603 were used as control materials.

For fungal isolates, the swab samples were cultured on Sabouraud Dextrose Agar and incubated at 28°C for 7 days to identify fungal species [19]. Identification was done by cultural characteristics and tease mount using lactophenol cotton blue stain. Macroscopically, an initial white cottony mycelium, which later turned orange‐pink to reddish orange, was recorded. There was fluffy aerial mycelium with a powdery surface. Microscopic examination revealed septate hyphae and macroconidia. Septa have central spores, suggestive of Neurospora. A macroscopic appearance of *Fusarium solani* revealed initial white cottony growth, which later turns cream to gray with bluish‑green or brownish reverse. On microscopy, septate hyphae and abundant curved or sickle‐shaped macroconidia were present. Oval or cylindrical microconidia were also present, together with chlamydospores were also revealed. All microbiological media and biochemical reagents used in this study were manufactured by Oxoid (United Kingdom).

## 3 Statistical Analysis

Data were summarized using descriptive statistics, including frequencies and percentages of microbial isolates by surface type and sampling week.

## 4 Results

A total of 40 swabs were collected from radiology equipment, accessories, and work surfaces. Thirty‐five samples (87.5%) yielded microbial growth. Four distinct bacterial species were identified: coagulase‐negative Staphylococci (CoNS), *Klebsiella pneumoniae*, *Bacillus* species, and *Staphylococcus aureus*, across four surface types. Coagulase‐negative Staphylococci (CoNS) accounted for the majority of bacterial isolates (*n* = 6; 66.7%), followed by *K. pneumoniae* (*n* = 1; 11.1%), *Bacillus spp.* (*n* = 1; 11.1%), and *S. aureus* (*n *= 1; 11.1%). The 35/43 cm image receptor exhibited the highest bacterial contamination, yielding four of the nine total isolates (44.4%). Details of bacterial occurrence across equipment and surfaces are presented in Table 2, while weekly distribution and colony‐forming unit (CFU) counts are summarized in Table 3.

**Table 2:** Bacterial isolates across radiology equipment, accessories, and surfaces.

	Gram‐negative species				
Bacterial isolates	Total *n* = 9	35/43 cm Image receptor *n* = 4	X‐ray tube head handle *n* = 3	Horizontal Bucky surface *n* = 1	Erect Bucky surface *n* = 1
*Klebsiella pneumoniae*	1 (11.1%)	1 (25.0%)	0 (0.0%)	0 (0.0%)	0 (0.0%)
Gram‐positive species					
Coagulase‐negative Staphylococci	6 (66.7%)	2 (50.0%)	2 (66.7%)	1 (100.0%)	1 (100.0%)
*Bacillus* species	1 (11.1%)	1 (25.0%)	0 (0.0%)	0 (0.0%)	0 (0.0%)
*Staphylococcus aureus*	1 (11.1%)	0 (0.0%)	1 (33.3%)	0 (0.0%)	0 (0.0%)

**Table 3:** Bacterial isolates and their colony counts across radiology equipment, accessories, and surfaces.

Week	Radiology equipment/accessories/surfaces	Organism found	Colony count (CFU/cm^2^)
1	35/43 cm Image receptor	*Klebsiella pneumoniae*	1.25 × 10^6^
2	35/43 cm Image receptor	Coagulase‐negative Staphylococci	2.95 × 10^6^
	X‐ray tube head handle	*Staphylococcus aureus*	6.5 × 10^5^
3	35/43 cm Image receptor	Coagulase‐negative Staphylococci	2.6 × 10^5^
	Horizontal Bucky surface	Coagulase‐negative Staphylococci	Statistically non‐ significant colonies (< 30 colonies)
	X‐ray tube head handle		
4	35/43 cm Image receptor	*Bacillus spp*.	Statistically non‐ significant colonies (< 30 colonies)
	Erect Bucky surface	Coagulase‐negative Staphylococci	2.95 × 10^5^
	X‐ray tube head handle		6.0 × 10^6^

Fungal growth was observed in 26 of 40 samples (65%). Two genera, *Neurospora* and *Fusarium*, were isolated across all 10 items, with *F. solani* predominance (*n* = 16; 61.5%), while *Neurospora* species appeared in 10 samples (38.5%). Table 4 presents the distribution of fungal isolates by equipment type.

**Table 4:** Fungal isolates across radiology equipment, accessories, and surfaces.

Radiology equipment/accessories/surfaces	Total *n* = 26	*Neurospora* species *n* = 10	*Fusarium solani* *n* = 16
24/30 cm Image receptor	3 (11.54%)	0 (0.0%)	3 (18.75%)
35/43 cm Image receptor	2 (7.69%)	2 (20.00%)	0 (0.0%)
Exposure button	3 (11.54%)	1 (10.00%)	2 (12.50%)
Main door handle	3 (11.54%)	0 (0.0%)	3 (18.75%)
Erect Bucky handle	2 (7.69%)	0 (0.0%)	2 (12.50%)
Erect Bucky surface	3 (11.54%)	2 (20.00%)	1 (6.25%)
Horizontal Bucky surface	2 (7.69%)	1 (10.00%)	1 (6.25%)
Horizontal Bucky handle	3 (11.54%)	2 (20.00%)	1 (6.25%)
X‐ray tube head handle	3 (11.54%)	1 (10.0%)	2 (12.50%)
Tube head collimator knob	2 (7.69%)	1 (10.0%)	1 (6.25%)

## 5 Discussion

This study investigated the presence of microorganisms on 10 radiology equipment surfaces and accessories at a major hospital in the Volta Region of Ghana. The sampled items included the main door handle, erect and horizontal Bucky handles and surfaces, tube head collimator knob, x‐ray tube head handle, exposure button, and two image receptors. Four of the 10 swabbed equipment, accessories and surfaces (Table 2) yielded four different bacterial isolates (*Klebsiella pneumoniae*, coagulase‐negative Staphylococci, *Bacillus* species, and *Staphylococcus aureus)*. These findings are consistent with earlier reports documenting similar isolates from radiology environments in both high‐ and low‐income settings [17, 22]. The 35/43 cm image receptor exhibited the highest frequency of bacterial contamination (4 of 9 isolates, 44.4%), with a maximum colony count of 4.5 × 10^5^ CFU/cm^2^. This finding is consistent with Kim et al. [7], who similarly identified the image receptor as a significant reservoir of bacterial contamination. The high burden of microbes on image receptors may be attributed to frequent handling and direct skin contact during radiographic procedures, particularly among high‐risk or hospitalized patients. Inadequate cleaning and disinfection practices may also contribute to the persistence of viable organisms on such high‐touch devices [17]. Despite daily cleaning and additional cleaning of visibly soiled surfaces, contamination was still detected on high‐touch radiology equipment. Since sampling was conducted during routine working hours, these findings likely reflect contamination accumulating during use. This underscores the need for disinfection between patient examinations, not just once‐daily cleaning.

*Klebsiella pneumoniae* was detected on the 35/43 cm image receptor. This Gram‐negative pathogen is commonly implicated in hospital‐acquired pneumonia, urinary tract infections, and septicemia, and is notable for multidrug resistance [22]. Patients who suffer from chronic pneumonia caused by *K. pneumoniae* may visit the radiology department for chest x‐ray examinations and could therefore transmit these bacteria to image receptors if they come in contact with them. The *K. pneumoniae*‐contaminated image receptor may serve as a vehicle for transmission of the organism, especially to immunocompromised patients and other healthcare professionals. This becomes a burden for the already strained healthcare system because hospital‐acquired pneumonia can be difficult to treat due to the resistance of *K. pneumoniae* to various antibiotics [23]. *Bacillus spp.* was also found on the 35/43 cm image receptor. Although *Bacillus spp*. is usually considered a non‐pathogenic microbe, it is known to cause septicemia in immunocompromised patients. The clinical implication of having *Bacillus spp*. on an image receptor is that it poses as a fomite for cross‐infection for high‐risk patients, such as those admitted in the intensive care units because these image receptors are frequently used in mobile x‐ray examinations and come in contact with these patients [16].

Furthermore, Coagulase‐Negative Staphylococci were the most common bacterial isolates and were present on the swabbed 35/43 cm image receptor, x‐ray tube head handle, horizontal Bucky surface, and the erect Bucky surface (Tables 2 and 3). This could pose health risks to patients and staff due to frequent contact with contaminated equipment during procedures. Coagulase‐negative Staphylococci (CoNS), which are associated with laryngological infections such as rhinosinusitis, tonsillitis, and nasal skin infections, were detected on radiology equipment and accessories, potentially increasing the risk of infection among radiographers and patients [24]. Similarly, *Staphylococcus aureus* was detected on the x‐ray tube head handle and may cause furuncles, skin infections, postoperative wound infections, and pneumonia [25]. This is particularly concerning given the high antimicrobial resistance of *S. aureus*, which can complicate treatment [7].

Two fungal isolates—*F. solani* and *Neurospora* species—were also present across the 10 items of radiology equipment, accessories, and surfaces swabbed in our study (Table 4). These isolates differ considerably in clinical relevance. *Fusarium solani*'s detection on equipment surfaces in this study reflects environmental contamination and the organism's capacity to persist on inanimate surfaces, serving as a reservoir from which infection could plausibly arise among high‐risk patients. *Neurospora* species, on the other hand, are ubiquitous environmental saprophytes with no established role as human pathogens [26]. It serves as an indirect marker of environmental hygiene conditions, such as inadequate ventilation, air filtration, or dust control within the department [27]. Their detection highlights the need for effective environmental hygiene and routine cleaning and disinfection of radiology equipment and surfaces to minimize potential microbial reservoirs within the department.

Most of the radiological equipment, accessories, and surfaces, such as the erect Bucky surface and the 35 × 43 cm image receptor, were heavily contaminated. Low or statistically non‐significant bacterial growth was observed for *Bacillus* species and Coagulase‐Negative Staphylococci on the 35 × 43 cm image receptor and the horizontal Bucky surface and x‐ray tube head handle, respectively. The contamination of this equipment poses a serious health risk to both staff and patients, particularly immunocompromised individuals.

A previous study has proposed different bacteriological standards for assessing clinical surface hygiene in hospitals. Dancer [28] suggested that the presence of *Staphylococcus aureus*, including methicillin‐resistant *Staphylococcus aureus* (MRSA), *Clostridium difficile*, vancomycin‐resistant enterococci, and various Gram‐negative bacilli indicates a need for enhanced cleaning practices. The second standard, suggesting increased cleaning and disinfection, is the quantitative aerobic colony count of < 5 CFU/cm^2^ on frequently touched surfaces in hospitals.

The estimated bacterial load observed in the current study was extremely high compared with those reported in previous studies. In Palestine, a microbial load ranging from 1.2 to 20 CFU/cm^2^ was detected on various items tested in the radiology department of a tertiary hospital [29]. Similarly, a study conducted in Sweden reported a maximum bacterial load of 20 CFU/cm^2^ on computed tomography and magnetic resonance imaging equipment [30]. The high burden of microbial contamination observed in our study supports the need for routine cleaning and disinfection practices using the appropriate disinfectant at the correct concentration, particularly for frequently touched equipment.

Taken together, these findings suggest that contamination in this department is concentrated on portable, patient‐shared equipment rather than distributed evenly across all surfaces, pointing to a specific, addressable target for infection control efforts rather than a generalized hygiene failure. For practice, this indicates a need for disinfection protocols that explicitly target portable, frequently reused items like image receptors between patients, rather than relying on broader cleaning schedules typically focused on fixed surfaces.

## 6 Limitations of the Study

This study has some limitations that should be considered when interpreting the findings. First, identification of bacterial and fungal isolates was based solely on macroscopic characteristics, and biochemical testing and microscopy using lactophenol cotton blue for bacterial and fungal identifications, respectively. No molecular testing was done. While these phenotypic approaches are routinely used in basic microbiological studies, they may lack sufficient specificity, particularly for closely related species, and thus misidentification cannot be completely excluded. Secondly, quality control procedures were performed only for bacterial isolates using standard reference strains, whereas no corresponding quality control measures were implemented for fungal identification. This may affect the reproducibility and accuracy of the fungal findings. Additionally, due to the limited sample size, inferential statistical analyses were not performed. A disinfectant‐neutralizing agent was not used prior to surface swabbing. As a result, residual disinfectants may be present on sampled surfaces. The effect of this disinfectant residue may have continued to exert antimicrobial activity during sample collection and transport, potentially reducing the recovery of viable microorganisms and leading to an underestimation of the actual microbial burden on the surfaces. Finally, this was a single‐center study, limiting its generalizability.

## 7 Conclusion

This study demonstrated microbial contamination of radiology equipment, accessories, and work surfaces, including the detection of potentially pathogenic bacterial and fungal organisms. The detection of both bacterial and fungal species on high‐touch items highlights the need for strict adherence to cleaning, disinfection, and hand hygiene protocols within radiology departments. Strengthening infection‐prevention and control practices will improve the safety of both patients and healthcare workers.

## 8 Recommendations for Clinical Practice and Future Research

- Implement routine environmental surveillance and microbial audits in radiology departments.
- Develop and enforce standardized cleaning and disinfection protocols based on international infection prevention and control guidelines.
- Future studies with larger sample sizes to allow for comparative and inferential analyses. Conduct national or multi‐center studies to characterize microbial contamination patterns across radiology facilities in Ghana.
- Provide continuous infection prevention and control training and compliance monitoring for radiology staff.
- Incorporate periodic antimicrobial resistance testing of isolates to inform evidence‐based infection control strategies.

## Author Contributions

**Seth Kwadjo Angmorterh:** conceptualization (lead), methodology (lead), data analysis (lead), manuscript writing – original draft (lead), manuscript writing – review and editing (equal). **John Gameli Deku:** conceptualization (supporting), methodology (supporting), manuscript writing – original draft (supporting). **Emmanuel Akyea‐Mensah, Patience Nyamekye Agyemang**, and **Nathaniel Awentiirin Angaag:** conceptualization (supporting), methodology (supporting), data analysis (supporting), manuscript writing – review and editing (supporting). **Riaan van de Venter, Hubert Amu, Sonia Aboagye, Jevas Omweri Kenyanya, Kafui Kossi Kekessie**, and **Benjamin Klugah‐Brown:** manuscript writing – review and editing (supporting). All authors have read and approved the final version of the manuscript.

## Funding

The authors have nothing to report.

## Conflicts of Interest

The authors declare no conflicts of interest.

## Transparency Statement

The lead author, Seth Kwadjo Angmorterh, affirms that this manuscript is an honest, accurate, and transparent account of the study being reported; that no important aspects of the study have been omitted; and that any discrepancies from the study as planned (and, if relevant, registered) have been explained.

## Data Availability

The authors confirm that the data supporting the findings of this study are available within the article.
